# The National Landscapes of Gastric Mucosa-Associated Lymphoid Tissue Lymphoma: Stable Trends in Black Populations and Late-Stage Tumors

**DOI:** 10.3390/cancers16112024

**Published:** 2024-05-27

**Authors:** Yazan Abboud, Charlotte Pirquet, Kiley Timmons, Ibrahim Abboud, Mina Awadallah, Ahmed Al-Khazraji, Kaveh Hajifathalian

**Affiliations:** 1 Department of Internal Medicine, Rutgers New Jersey Medical School, Newark, NJ 07103, USA; cep145@njms.rutgers.edu (C.P.); kiley.timmons@rutgers.edu (K.T.); 2 School of Medicine, University of California Riverside, Riverside, CA 92521, USA; brhoum.abboud@gmail.com; 3 Division of Gastroenterology and Hepatology, Rutgers New Jersey Medical School, Newark, NJ 07103, USA; mina.awadallah@hotmail.com (M.A.); aa2758@njms.rutgers.edu (A.A.-K.)

**Keywords:** gastric cancer, gastric mucosa-associated lymphoid tissue (MALT) lymphoma, MALTOMA, disparities, epidemiology, incidence, mortality, outcomes

## Abstract

**Simple Summary:**

*Helicobacter pylori* (*H. Pylori*) eradication has been the mainstream for preventing and treating gastric mucosa-associated lymphoid tissue (MALT) lymphoma. Prior studies have shown variations in the eradication rates of *H. Pylori* in the US. This can influence gastric MALT lymphoma occurrence and outcomes. Therefore, we conducted a comprehensive analysis of incidence and mortality rates of gastric MALT lymphoma in different demographic-specific populations. We found that gastric MALT lymphoma incidence rates have been decreasing in the US over the past two decades. This decline was observed in both sexes, various age groups, and all race/ethnic populations except Black individuals. The decrease was also seen in early-stage tumors but not in late-stage tumors. Mortality analysis showed decreasing mortality in males, older adults, early-stage tumors, and most race/ethnic groups but not in females, younger adults, Black individuals, or late-stage tumors, which had non-decreasing rates. Our findings hold important public health implications and encourage further investigations of gastric MALT lymphoma risk factors, including disparities in *H. Pylori* screening, management, and outcomes.

**Abstract:**

Background: *Helicobacter pylori* (*H. Pylori*) eradication has been the mainstream for preventing and treating gastric mucosa-associated lymphoid tissue (MALT) lymphoma. Prior data showed disparities in eradication rates of *H. Pylori* between different populations. This can potentially impact the occurrence of gastric MALT lymphoma. There are limited data on the incidence and mortality rates and trends of gastric MALT lymphoma in the US. Therefore, the aim of the current study was to conduct a time-trend analysis of gastric MALT lymphoma incidence and mortality rates in different populations. Methods: The incidence rates of gastric MALT lymphoma were calculated from the United States Cancer Statistics (USCS) database (which covers nearly 98% of the US population) between 2001–2020 and were age-adjusted to the standard 2000 US population using SEER*Stat software (version 8.4.3, national cancer institute “NCI”). Incidence-based mortality (IBM) rates, also age-adjusted to the standard 2000 US population, were calculated from the Surveillance Epidemiology and End Results (SEER) database. Tumor location was specified using ICD-O-3 codes C 160–C 169 with malignant behavior. Histopathology was specified using the ICD-O-3 code 9699. The rates were categorized by sex, age, race/ethnicity, and tumor stage at diagnosis. Age groups were older adults (aged 55 years or older) and younger adults (aged younger than 55 years). Race/ethnic groups included Non-Hispanic White (White), Non-Hispanic Black (Black), Hispanic, Non-Hispanic Asian/Pacific Islander (API), and Non-Hispanic American Indian/Alaska Native (AI/AN), as reported in the database. Stage at diagnosis included early stage (in situ and localized tumors) and late stage (regional and distant site tumors). Joinpoint Regression Software (version 5.0.2, NCI) using the weighted Bayesian Information Criteria method was used to generate time trends. Trends were reported as annual percentage change (APC) and average APC (AAPC). Parametric estimations were used with a two-sided *t*-test to evaluate the trends with a *p*-value cutoff at 0.05. Results: There were 21,625 patients diagnosed with gastric MALT lymphoma in the US between 2001 and 2020. Overall, incidence rates were significantly decreasing over the study period (AAPC = −1.93). This decrease was seen in males (AAPC = −1.67) and in females (AAPC = −1.66) (Figure). When categorized by age groups, older adults also experienced a significant decrease in gastric MALT lymphoma incidence rates (AAPC = −1.66). While this was also seen in younger adults, the rates were decreasing at a slower pace (AAPC = −1.38). When categorizing the trends by race/ethnicity, incidence rates were significantly decreasing in White (AAPC = −2.09), Hispanic (AAPC = −1.61), and API (AAPC = −3.92) populations. However, the rates were stable among Blacks. While early-stage tumors experienced a significant decrease (AAPC = −1.10), the rates were stable for late-stage tumors. When evaluating mortality, there were 11,036 patients whose death was attributed to gastric MALT lymphoma between 2000 and 2020. IBM rates were decreasing in males (AAPC = −1.47), older adults (AAPC = −1.55), Whites (AAPC = −1.23), Hispanics (AAPC = −1.73), APIs (AAPC = −2.30), and early-stage tumors (AAPC = −1.08). On the other hand, IBM rates were stable in females, younger adults, Blacks, and late-stage tumors. Discussion: An extensive nationwide data analysis encompassing nearly 98% of patients diagnosed with gastric MALT lymphoma in the US unveils a declining trend in the incidence of cancer overall over the past two decades. This decline is observed in both sexes and various age groups. When stratifying by race and ethnicity, this incidence has been decreasing in all populations except among Black individuals. While early-stage tumors have also demonstrated a significant decrease in incidence rates, late-stage tumors have shown no parallel decline. Mortality evaluation also revealed an improvement in most of the US population except among females, younger adults, Black individuals, and late-stage tumors. While the cause of our findings is unclear, it could be driven by disproportionate exposure to risk factors, including *H. Pylori*, and disparities in screening, management, and outcomes. Future studies are warranted to investigate factors contributing to worse outcomes of gastric MALT lymphoma, especially in the Black population.

## 1. Introduction 

Gastric mucosa-associated lymphoid tissue (MALT) lymphoma, a subtype of extranodal marginal zone lymphoma, predominantly emerges in the stomach, primarily due to inflammation-triggered neoplastic changes. Extranodal MALT lymphoma comprises up to 8% of all non-Hodgkin lymphomas, and most cases occur in the stomach [1,2]. The etiology of primary gastric involvement, estimated at 34% of all primary sites of extranodal marginal zone lymphomas, varies among different populations and offers avenues for focused treatment [3].

Among extranodal MALT lymphomas, the unique etiology of gastric MALT lymphoma allows for focused treatment. The pathogenesis of MALT lymphoma is due to a multistep process involving chronic antigen stimulation, host genetics, and likely other environmental and individual factors [4]. More specifically, primary gastric diseases are usually acquired due to localized *Helicobacter pylori (H. pylori)* infection (i.e., gastritis), which results in chronic inflammation and ultimately leads to the accumulation of genetic mutations in B-cells [5]. *H. pylori* is very common, colonizing the gut flora of more than 50% of individuals worldwide, albeit with significant differences in prevalence based on geographic location [6]. Due to its close association with the development of gastric MALT lymphoma, *H. pylori* infection has become the predominant treatment target over the past several decades. Eradication of *H. pylori* has consistently demonstrated high remission rates and is associated with better outcomes for patients with gastric MALT lymphoma [7,8,9]. However, much of the prior data have shown disparities in eradication rates of *H. pylori* between various populations, with discrepancies apparent in global geographic location and race, often necessitating different medication regimens or treatment strategies [10,11,12]. Given the demonstrated importance of *H. pylori* eradication in treatment and the variations in the eradication rates among different populations, this might impact the prevalence of MALT lymphoma in different groups.

Despite gastric MALT lymphoma representing a notable subset of extranodal MALT lymphomas, research on its incidence remains limited. There is a dearth of literature exploring gastric MALT lymphoma trends, with recent data specific to the US especially lacking. Given the varying risk factors driving gastric MALT lymphoma across populations, it is anticipated that time trends will differ based on these factors. Understanding demographic-based trends holds practical significance, as it could inform modifications to targeted treatment strategies. Therefore, the aim of the current study was to conduct a comprehensive analysis of gastric MALT lymphoma incidence and mortality rates and trends categorized by different demographic and tumor-specific populations.

## 2. Methods

This is an analysis of the age-adjusted incidence and incidence-based mortality rates of gastric MALT lymphoma in different demographic populations in the US between 2001 and 2020.

For this study, incidence data between 2001 and 2020 were collected from the United States Cancer Statistics (USCS) database. The USCS is the official source of federal cancer incidence statistics in the US and covers nearly 98% of the US population. The USCS database combines data from two nationwide databases; the first one is the Surveillance Epidemiology and End Results (SEER) database, and the second is the National Program of Cancer Registries (NPCR) database. Each database collects data from certain US states and feeds it to the USCS database to cover nearly 98% of the population [13]. Thereafter, these data undergo quality checks and review to maintain high quality and standardization [14]. Incidence-based mortality (IBM) rates between 2000 and 2020 were collected from the SEER 22 database. IBM is the mortality rate for patients with known incidence and time of cancer diagnosis. It allows for the categorization of mortality rates by different variables, such as age at diagnosis, sex, race/ethnicity, and stage at diagnosis.

Gastric MALT lymphoma incidence rates per 100,000 population were obtained and age-adjusted to the standard 2000 US population using SEER*Stat software (version 8.4.3, national cancer institute “NCI”). Tumor location was specified as the “Primary Site” variable using the following codes: C 160–C 169 with malignant behavior. Histopathological identification was accomplished using the International Classification of Diseases for Oncology, Third Edition, Site Record ICD-O-3/WHO 2008 code 9699, as performed in prior published studies [15]. Incidence rates of gastric MALT lymphoma were categorized by sex, age, race/ethnicity, and stage at diagnosis. Age groups were older adults (patients aged 55 years or older) and younger adults (patients aged younger than 55 years). Race/ethnic groups included Non-Hispanic White (White), Non-Hispanic Black (Black), Hispanic, Non-Hispanic Asian/Pacific Islander (API), and Non-Hispanic American Indian/Alaska Native (AI/AN). Stage at diagnosis included early stage (in situ and localized tumors) and late stage (regional and distant site tumors).

Gastric MALT lymphoma IBM rates per 100,000 population were obtained and age-adjusted to the standard 2000 US population using SEER*Stat software. Tumors were identified using the same codes as the incidence data. IBM rates were also categorized by sex, age, race/ethnicity, and tumor stage at diagnosis, in a similar methodology to the incidence analysis. For stage at diagnosis, mortality rates were collected between 2004 and 2020 based on the availability of the data.

Thereafter, we analyzed time trends for incidence and IBM rates to estimate the annual percentage change (APC) and average annual percentage change (AAPC). This analysis was conducted via Joinpoint Regression Software (version 5.0.2, NCI) and the weighted Bayesian Information Criteria “BIC” method, which is a statistical methodology utilized to generate trends over time [16,17,18]. Parametric estimations were used with a two-sided *t*-test to evaluate the trends, with a *p*-value cutoff of 0.05 for statistical significance.

## 3. Results

### 3.1. Gastric MALT Lymphoma Demographics and Overall Incidence Rates and Trends

Between 2001 and 2020, 21,625 individuals in the US were diagnosed with gastric MALT lymphoma. The majority were female (50.6%), aged over 55 (80.1%), and of Non-Hispanic White race/ethnicity (71.2%). A significant portion of diagnoses occurred at an early stage (69.9%). Over the study period, there was a notable decline in the incidence rates of gastric MALT lymphoma per 100,000 population, decreasing from 0.33 in 2001 to 0.23 in 2020 (AAPC = −1.93, 95% CI −3.18–−0.66) (Table 1).

### 3.2. Gastric MALT Lymphoma Incidence Trend per Sex

**Figure 1:**
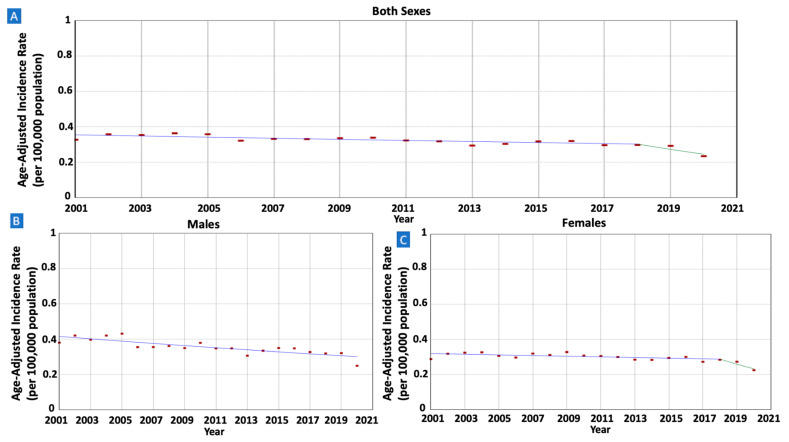
Time trends and age-adjusted incidence rates per 100,000 population for gastric mucosa-associated lymphoid tissue (MALT) lymphoma among different groups categorized by sex. (**A**): Both Sexes, (**B**): Males, (**C**): Females.

The incidence rates of gastric MALT lymphoma per 100,000 population exhibited a decline among males, dropping from 0.38 in 2001 to 0.25 in 2020 (AAPC = −1.67, 95% CI −2.24–−1.09). A similar trend was observed in females, with rates decreasing from 0.29 in 2001 to 0.23 in 2020 (AAPC = −1.66, 95% CI −3.02–−0.28). (Refer to Figure 1 and Table 1).

### 3.3. Gastric MALT Lymphoma Incidence Rates and Trends per Age

**Figure 2:**
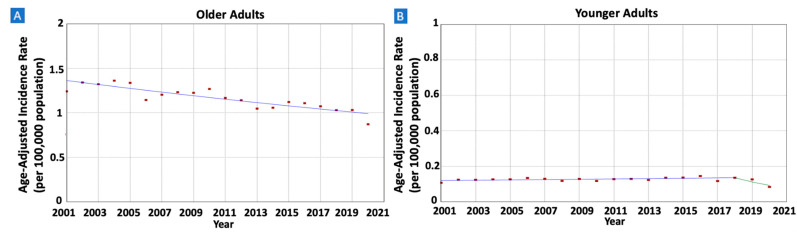
Time trends and age-adjusted incidence rates per 100,000 population for gastric mucosa-associated lymphoid tissue (MALT) lymphoma among different groups categorized by age. (**A**): Older Adults, (**B**): Younger Adults.

When stratified by age groups, older adults exhibited a notable decrease in gastric MALT lymphoma incidence rates, declining from 1.24 in 2001 to 0.87 in 2020 (AAPC = −1.66, 95% CI −2.13–−1.18) (see Figure 2). Similarly, albeit at a slower pace, younger adults also experienced declining rates from 0.11 in 2001 to 0.08 (AAPC = −1.38, 95% CI −2.24–−0.46).

### 3.4. Gastric MALT Lymphoma Incidence Rates and Trends per Race/Ethnicity

**Figure 3:**
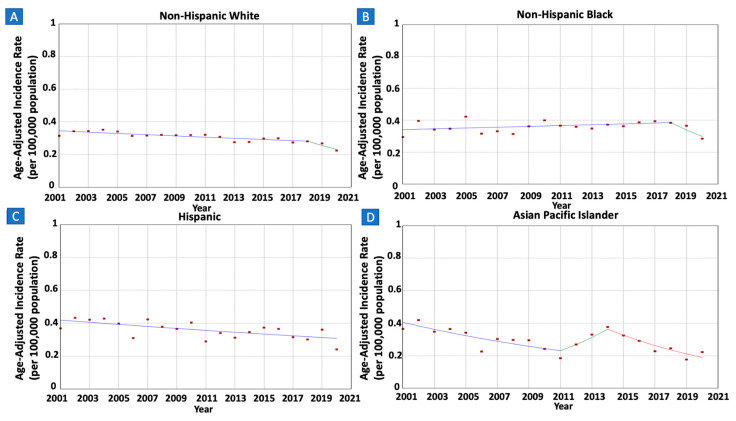
Time trends and age-adjusted incidence rates per 100,000 population for gastric mucosa-associated lymphoid tissue (MALT) lymphoma among different groups categorized by race/ethnicity. (**A**): Non-Hispanic White, (**B**): Non-Hispanic Black, (**C**): Hispanic, (**D**): Asian Pacific Islander.

When analyzing trends by race/ethnicity, incidence rates displayed significant decreases among White individuals, dropping from 0.31 in 2001 to 0.22 in 2020 (AAPC = −2.09, 95% CI −2.72–−1.30), Hispanic individuals, from 0.37 in 2001 to 0.24 in 2020 (AAPC = −1.61, 95% CI −2.70–−0.32), and API individuals, from 0.37 in 2001 to 0.22 in 2020 (AAPC = −3.92, 95% CI −5.14–2.96) (refer to Figure 3). However, rates remained stable among Black individuals (AAPC −0.71, 95% CI −1.67–0.80). Insufficient cases were reported among AI/AN individuals in at least one given calendar year, hindering trend estimation.

### 3.5. Gastric MALT Lymphoma Incidence Rates and Trends per Tumor Stage at Diagnosis

**Figure 4:**
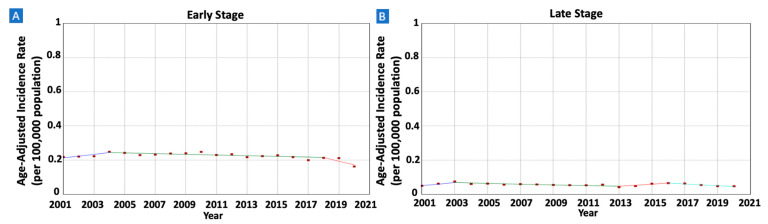
Time trends and age-adjusted incidence rates per 100,000 population for gastric mucosa-associated lymphoid tissue (MALT) lymphoma among different groups categorized by tumor stage at diagnosis. (**A**): Early-Stage Tumors, (**B**): Late-Stage Tumors.

When stratified by tumor stage at diagnosis, early-stage tumors exhibited a notable decrease from 0.22 in 2001 to 0.16 in 2020 (AAPC = −1.10, 95% CI −1.84 to −0.31). Conversely, rates remained stable for late-stage tumors (AAPC = −0.56, 95% CI −1.70–0.53). (Table 1 and Figure 4).

### 3.6. Gastric MALT Lymphoma IBM Rates and Trends

**Figure 5:**
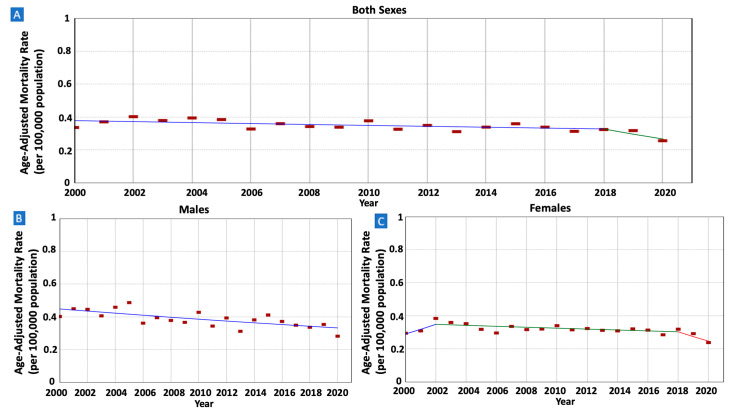
Time trends and age-adjusted incidence-based mortality rates per 100,000 population for gastric mucosa-associated lymphoid tissue (MALT) lymphoma among different groups categorized by sex. (**A**): Both Sexes, (**B**): Males, (**C**): Females.

**Figure 6:**
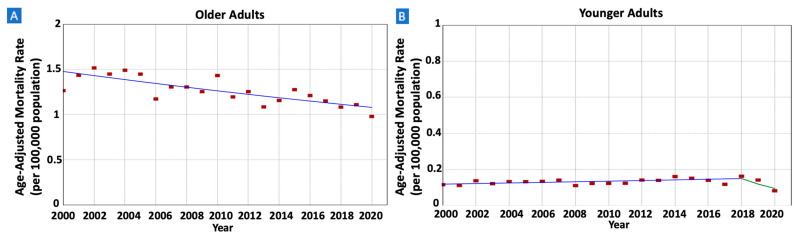
Time trends and age-adjusted incidence-based mortality rates per 100,000 population for gastric mucosa-associated lymphoid tissue (MALT) lymphoma among different groups categorized by age. (**A**): Older Adults, (**B**): Younger Adults.

**Figure 7:**
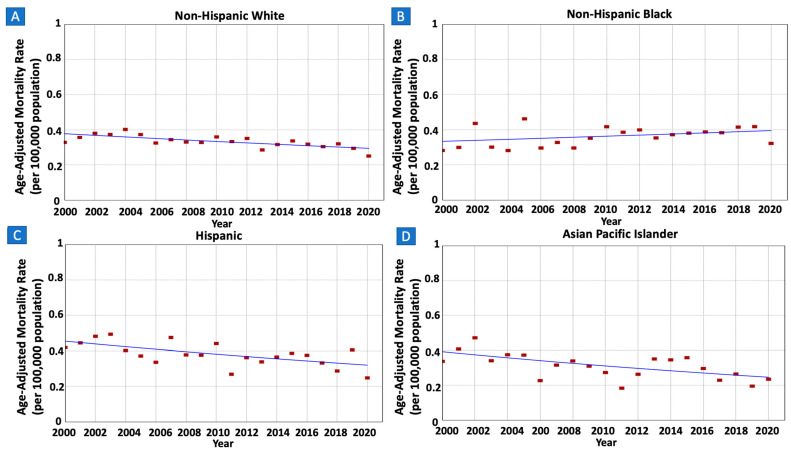
Time trends and age-adjusted incidence-based mortality rates per 100,000 population for gastric mucosa-associated lymphoid tissue (MALT) lymphoma among different groups categorized by race/ethnicity. (**A**): Non-Hispanic White, (**B**): Non-Hispanic Black, (**C**): Hispanic, (**D**): Asian Pacific Islander.

**Figure 8:**
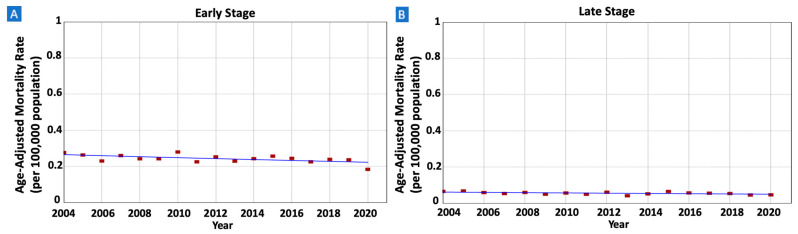
Time trends and age-adjusted incidence-based mortality rates per 100,000 population for gastric mucosa-associated lymphoid tissue (MALT) lymphoma among different groups categorized by tumor stage at diagnosis. (**A**): Early-Stage Tumors, (**B**): Late-Stage Tumors.

There were 11,036 patients whose death was attributed to gastric MALT lymphoma in the SEER database between 2000 and 2020. IBM rates were decreasing in males (AAPC = −1.47, 95% CI −2.22–−0.69) but not in females (AAPC = 0.80, 95% CI −1.63–0.15) (Figure 5 and Table 2). The rates were also decreasing in older adults (AAPC = −1.55, 95% CI −2.13–−0.97) but not in younger adults (AAPC = −1.12, 95% CI −2.96–1.34) (Figure 6). When stratified by race/ethnicity, IBM rates were decreasing in Whites (AAPC = −1.23, 95% CI −1.85–−0.65), Hispanics (AAPC = −1.73, 95% CI −2.99–−0.33), and APIs (AAPC = −2.30, 95% CI −3.66–−0.73). However, the rates were stable in Blacks (AAPC = 0.85, 95% CI −0.33–2.16) (Figure 7). When categorized by stage at diagnosis, IBM rates were decreasing in early-stage tumors (AAPC = −1.08, 95% CI −1.89–−0.25) but not in late-stage tumors (AAPC = −1.31, 95% CI −2.81–0.33) (Figure 8).

## 4. Discussion

An extensive nationwide data analysis encompassing nearly 98% of patients diagnosed with gastric MALT lymphoma in the US unveils a declining trend in cancer incidence overall over the past two decades. This decline is observed in both sexes and various age groups. When stratifying by race and ethnicity, the incidence has been decreasing in all populations with the exception of Black individuals. While early-stage tumors have also demonstrated a significant decrease in incidence, late-stage tumors have shown no parallel decline. When evaluating mortality, the rates were decreasing in males, older adults, early-stage tumors, and most race/ethnic groups, but not in females, younger adults, Black individuals, or late-stage tumors.

Prior research and literature for nationwide data investigating incidence rates and trends of gastric MALT lymphoma are sparse. A prior nationwide analysis of the SEER 18 database, which covers 27.8% of the US population, showed that the age-adjusted incidence rates of gastric MALT lymphoma decreased between 2000 and 2018 [19]. As for sex-specific differences, most of the literature has found similar incidence rates of gastric MALT lymphoma in both sexes, with variations in studies finding a slight predominance in males or females [20,21,22,23]. Our study findings echo these results, demonstrating approximately similar incidence rates between males and females. However, when evaluating the trends over time, there is very limited data on sex-specific trends. Our study adds to the existing literature and demonstrates that the incidence rates of gastric MALT lymphoma were decreasing at a similar rate in men (AAPC = −1.66) and women (AAPC = −1.67). These findings help inform policymakers of efficient diagnosis and management modalities of this malignancy in the overall US population.

When assessing the incidence rates of gastric MALT lymphoma by age groups, our findings align with previous observations, showcasing a significant decrease in incidence rates among older adults, while the decline among younger adults is more gradual. A prior study by Rustgi et al. analyzed the incidence of this malignancy between 2001–2018 and showed a significantly decreasing incidence in older adults, but this was not seen amongst younger adults [19]. Our study demonstrates a significant decrease in gastric MALT lymphoma incidence rates in older adults (AAPC = −1.66) and a slower decrease in younger adults (AAPC = −1.38). The differences between both studies are likely due to the larger sample size in our analysis and its extension over a longer and more recent time period. Furthermore, a smaller study in Italy between 1997 and 2007 found a bimodal distribution in incidence peaking at ages 30 and 70, with the younger group of patients more likely to be males with cutaneous involvement [24]. The authors suggest a potential role of infectious etiologies in the pathogenesis of gastric MALT lymphoma in younger adults, especially with the known association between *H. Pylori* gastritis and gastric MALT lymphoma. With that in mind, *H. Pylori* negative gastric MALT lymphomas are becoming more common as *H. Pylori* diagnosis and treatment have significantly improved since the 1990s [25]. Further investigation is warranted to understand if *H. Pylori*-negative cases or other infections-related etiologies are more prevalent among younger adults and responsible for the slower decline in the incidence of gastric MALT lymphoma in this subgroup.

Regarding race and ethnicity, while prior research hinted at a decreasing incidence across various populations, our study provides a more comprehensive examination. The previously mentioned study by Rustgi et al. conducted a nationwide analysis of the SEER 18 database and showed a decrease in the incidence rates of gastric MALT lymphoma in White, Hispanic, Black, and API populations [19]. While our study is consistent with most of their data, demonstrating a decrease in incidence rates of gastric MALT lymphoma in White, Hispanic, and API populations, we demonstrate stable rates among Black individuals. Possible explanations for this discrepancy include differences in *H. Pylori* prevalence and disparities in access to care and treatment outcomes. Our study covers a larger portion of the US population over a more recent time period (21,625 patients between 2001 and 2020) compared to the prior study (4688 patients between 2000 and 2018), possibly explaining the different results. It is also worth noting that the greatest decrease in incidence rates in all race/ethnic groups in our study was seen in the past few years, which was not analyzed in the prior study by Rustgi et al. The prevalence of *H. Pylori* is significantly higher in Black, Hispanic, and low socio-economic populations [26]. Furthermore, a big retrospective study of 371,813 US veteran patients between 1994 and 2018 showed that Black individuals with *H. Pylori* were twice as likely to develop gastric cancer compared to White individuals [27]. Given that early treatment and the successful eradication of *H. Pylori* are crucial in lowering the risk of malignancy and require multiple medications and office visits, it is reasonable to hypothesize that access to care and social determinants of health are contributing to the non-decreasing rates in the black population.

While *H. Pylori* treatment success rates between different races are approximately the same, Black patients undergo less eradication testing compared to other races, and they have a higher likelihood of failure to complete eradication tests [28,29,30]. Prior data also suggested that African Americans have lower clearance rates of *H. Pylori*, which may be due to genetic and/or socio-economic factors [12]. Moreover, African ancestry was shown to be associated with a higher risk of *H. Pylori* infection, regardless of education status, employment, or household income [31]. Further research is warranted to investigate the specific causes of these disparities in *H. Pylori* treatment, such as genetic predisposition, lack of access to care, misdiagnosis, medication nonadherence, or other socioeconomic factors. This can help lower the risk of developing gastric MALT lymphoma in the Black population.

Furthermore, our study underscores the impact of stage at diagnosis on incidence trends. There is limited research assessing gastric MALT lymphoma incidence trends per stage at diagnosis. A multicenter study in South Korea involving 1163 patients between 2000 and 2018 showed that 97.6% of those patients had the early-stage disease [32]. There appears to be no data assessing the US national temporal changes in this malignancy per its stage at diagnosis. This is essential given the variation in outcomes between early- and late-stage diseases. Our study illustrates that the overall decrease in the incidence of gastric MALT lymphoma is predominately driven by early-stage diseases. Most early diseases occur due to symptom presentation prompting endoscopic evaluation, which has been growing in the US over the last few decades. Additionally, access to healthcare influences diagnostic stages across different demographic groups. Social determinants of health profoundly impact early detection, particularly among underserved populations, demonstrating that barriers such as lack of insurance, cultural stigmas, and limited awareness can delay diagnosis. Identifying these factors is crucial for informing public health strategies to improve early detection and treatment, particularly in vulnerable communities.

Our evaluation of IBM rates allows for the categorization of mortality rates by different demographics and tumor characteristics. We show that there is a lack of mortality improvement among females, younger adults, Black individuals, and tumors diagnosed at a late stage. The non-decreasing mortality among Black individuals correlates with the incidence data and prompts future research to investigate the revealed disparities and identify factors contributing to worse outcomes among Black patients.

Strengths of our study include the utilization of most comprehensive US nationwide cancer incidence database and analyzing the largest sample size to date, with 21,625 patients diagnosed with gastric MALT lymphoma between 2001 and 2020. We also provide a comprehensive mortality analysis using the SEER 22 database with a big sample size of 11,036 deaths. We evaluate the interplay of several demographic and tumor-specific variables such as sex, age, race/ethnicity, and stage at diagnosis, highlighting disparities in the incidence of this malignancy across different populations. Furthermore, we used joinpoint regression and the modified BIC method for time-trend analysis given their reliability and flexibility, especially in such large datasets with big population sizes [17,33]. However, our study has several limitations, including a lack of clinical variables to assess the risk of gastric MALT lymphoma in different populations. The observational nature of our study allows for the assessment of the interplay of different populations and tumor characteristics with the hope of guiding healthcare policies toward further evaluation of the revealed disparities. Lastly, other possible limitations of the large database we utilized include the loss of records and miscoding issues [34]. With that in mind, as stated earlier, the USCS database undergoes a rigorous process of validation to maintain high quality and minimize errors [13].

Using nationwide data covering nearly all of the US population, we show that the overall incidence of gastric MALT lymphoma has been decreasing over the past two decades. While this was seen in multiple demographic and tumor-specific populations, the rates did not decrease in the Black population nor in late-stage tumors. Mortality evaluation also revealed improvements in most of the US population except among females, younger adults, Black individuals, and late-stage tumors. While the cause of our findings is unclear, it could be driven by a disproportionate exposure to risk factors, including *H. Pylori*, and disparities in its screening, management, and outcomes. Future studies are warranted to investigate the factors contributing to worse outcomes of gastric MALT lymphoma, especially in the Black population.

## Figures and Tables

**Table 1:** Time trends for gastric mucosa-associated lymphoid tissue (MALT) lymphoma incidence rates among different demographic-specific groups.

Demographic Variable	Cases (N = 21,625) ^a^	N in 2001 (2020)	Age-Adjusted Incidence Rate (Crude Rate) in 2001	Age-Adjusted Incidence Rate (Crude Rate) in 2020	Time Period	APC ^b^ (95% CI)	AAPC (95% CI)
	**Sex**						
*Both Sexes* *(All Ages)*	21,625 (100%)	913 (938)	0.33 (0.32)	0.23 (0.29)	2001–2018	−0.93 * (−1.36 to −0.50)	−1.93 * (−3.18 to −0.66)
					2018–2020	−10.01 (−20.75 to 2.20)	
*Males*	10,676 (49.4%)	458 (455)	0.38 (0.33)	0.25 (0.29)	2001–2020	−1.67 * (−2.24 to −1.09)	−1.67 * (−2.24 to −1.09)
*Females*	10,949 (50.6%)	455 (483)	0.29 (0.32)	0.23 (0.30)	2001–2018	−0.61 * (−1.07 to –0.14)	−1.66 * (−3.02 to −0.28)
					2018–2020	−10.17 (−21.78 to 3.17)	
	**Age**						
*Older Adults*	17,317 (80.1%)	737 (800)	1.24 (1.23)	0.87 (0.84)	2001–2020	−1.66 * (−2.13 to –1.18)	−1.66 * (−2.13 to –1.18)
*Younger Adults*	4297 (19.9%)	175 (138)	0.11 (0.11)	0.08 (0.08)	2001–2018	−0.73 * (0.16 to 1.54)	−1.38 * (−2.24 to −0.46)
					2018–2020	−17.64 * (−24.46 to −5.71)	
	**Race/Ethnicity**						
*White*	15,390 (71.2%)	700 (627)	0.31 (0.37)	0.22 (0.33)	2001–2018	−1.18 (−1.57 to 0.36)	−2.09 * (−2.72 to –1.30)
					2018–2020	−9.50 * (−15.13 to −1.97)	
*Black*	2493 (11.5%)	78 (121)	0.30 (0.23)	0.28 (0.29)	2001–2018	0.71 (−0.22 to 5.03)	−0.71 (−1.67 to 0.80)
					2018–2020	−11.97 (−20.35 to 0.08)	
*Hispanic*	2147 (9.9%)	74 (105)	0.37 (0.20)	0.24 (0.18)	2001–2020	−1.61 (−2.70 to −0.32)	−1.61 * (−2.70 to −0.32)
*API*	832 (3.8%)	30 (48)	0.37 (0.25)	0.22 (0.23)	2001–2011	−5.49 * (−9.99 to −3.23)	−3.92 * (−5.14 to 2.96)
					2011–2014	16.40 * (3.05 to 23.78)	
					2014–2020	−10.27 * (−15.49 to −7.12)	
*AI/AN*	104 (0.5%)	^	^				
	**Stage at Diagnosis**						
*Early Stage*	15,125 (69.9%)	609 (649)	0.22 (0.22)	0.16 (0.20)	2001–2004	4.67 * (0.10 to 13.29)	−1.10 * (−1.84 to −0.31)
					2004–2018	−0.88 * (−1.77 to −0.02)	
					2018–2020	−10.58 * (−16.20 to −3.36)	
*Late Stage*	3859 (17.8%)	139 (190)	0.05 (0.05)	0.05 (0.06)	2001–2003	16.33 * (1.66 to 30.12)	−0.56 (−1.70 to 0.53)
					2003–2013	−3.70 * (−9.97 to −2.67)	
					2013–2016	12.07 * (3.02 to 17.23)	
					2016–2020	−8.93 * (−16.39 to −5.24)	

Non-Hispanic White (White), Non-Hispanic Black (Black), Non-Hispanic Asian/Pacific Islander (API), and Non-Hispanic American Indian/Alaska Native (AI/AN). ^a^ Data are presented as count numbers followed by percentages of the count numbers from the total cases of gastric MALT lymphoma in the database. ^b^ Time trends were computed using Joinpoint Regression Program (v.5.1.0.0, NCI) with three maximum joinpoints allowed (4-line segments). * implies statistical significance. ^ indicates there were too few cases of gastric MALT lymphoma in at least one calendar year to estimate a trend.

**Table 2:** Time trends for gastric mucosa-associated lymphoid tissue (MALT) lymphoma incidence-based mortality rates among different demographic-specific groups.

Demographic Variable	Deaths (N = 11,036) ^a^	N in 2000 (2020)	Age-Adjusted Mortality Rate (Crude Rate) in 2000	Age-Adjusted Mortality Rate (Crude Rate) in 2020	Time Period	APC ^b^ (95% CI)	AAPC (95% CI)
	**Sex**						
*Both Sexes* *(All Ages)*	11,036 (100%)	423 (480)	0.34 (0.31)	0.26 (0.31)	2000–2018	−0.79 (−3.83 to 15.34)	−1.72 (−3.01 to 0.32)
					2018–2020	−9.68 (−21.93 to 0.06)	
*Males*	5479 (49.6%)	215 (240)	0.40 (0.32)	0.28 (0.31)	2000–2020	−1.47 * (−2.22 to −0.69)	−1.47 * (−2.22 to −0.69)
*Females*	5557 (50.4%)	208 (240)	0.30 (0.30)	0.24 (0.30)	2000–2002	9.56 * (0.49 to 19.02)	−0.80 (−1.63 to 0.15)
					2002–2018	−0.86 (−2.01 to 0.15)	
					2018–2020	−9.74 * (−16.16 to −1.63)	
	**Age**						
*Older Adults*	8762 (79.4%)	335 (412)	1.27 (1.26)	0.98 (0.93)	2000–2020	−1.55 * (−2.13 to −0.97)	−1.55 * (−2.13 to −0.97)
*Younger Adults*	2271 (20.6%)	88 (68)	0.12 (0.11)	0.08 (0.08)	2000–2018	1.28 * (0.31 to 6.29)	−1.12 (−2.96 to 1.34)
					2018–2020	−20.32 * (−35.07 to −0.34)	
	**Race/Ethnicity**						
*White*	7412 (67.2%)	313 (295)	0.33 (0.38)	0.25 (0.36)	2000–2020	−1.23 * (−1.85 to −0.65)	−1.23 * (−1.85 to −0.65)
*Black*	1176 (10.7%)	33 (62)	0.28 (0.21)	0.32 (0.32)	2000–2020	0.85 (−0.33 to 2.16)	0.85 (−0.33 to 2.16)
*Hispanic*	1611 (14.6%)	50 (76)	0.42 (0.19)	0.25 (0.19)	2001–2020	−1.73 * (−2.99 to −0.33)	−1.73 * (−2.99 to −0.33)
*API*	692 (6.3%)	21 (39)	0.34 (0.24)	0.24 (0.26)	2001–2020	−2.30 * (−3.66 to −0.73)	−2.30 * (−3.66 to −0.73)
*AI/AN*	38 (0.3%)	1 (2)	0.10 (0.11)	0.20 (0.21)	^		
	**Stage at Diagnosis**						
*Early Stage*	7282 (66.0%)	371 (185)	0.28 (0.26)	0.18 (0.22)	2004–2020	−1.08 * (−1.89 to −0.25)	−1.08 * (−1.89 to −0.25)
*Late Stage*	1681 (15.2%)	89 (42)	0.07 (0.06)	0.05 (0.06)	2004–2003	−1.31 (−2.81 to 0.33)	−1.31 (−2.81 to 0.33)

Non-Hispanic White (White), Non-Hispanic Black (Black), Non-Hispanic Asian/Pacific Islander (API), and Non-Hispanic American Indian/Alaska Native (AI/AN). ^a^ Data are presented as death numbers followed by percentages of the death numbers from the total deaths attributed to gastric MALT lymphoma in the database. ^b^ Time trends were computed using Joinpoint Regression Program (v.5.1.0.0, NCI) with three maximum joinpoints allowed (4-line segments). * implies statistical significance. ^ indicates there were too few deaths attributed to gastric MALT lymphoma in at least one calendar year to estimate a trend. For stage at diagnosis, the time period was 2004 to 2020.

## Data Availability

Data used in this study can be found on the United States Cancer Statistics (USCS) and Surveillance Epidemiology and End Results (SEER) websites.
